# IL-18 Inhibits Growth of Murine Orthotopic Prostate Carcinomas via Both Adaptive and Innate Immune Mechanisms

**DOI:** 10.1371/journal.pone.0024241

**Published:** 2011-09-15

**Authors:** Brian Wan-Chi Tse, Pamela Joan Russell, Matthias Lochner, Irmgard Förster, Carl Andrew Power

**Affiliations:** 1 Lowy Cancer Research Centre, University of New South Wales, Sydney, New South Wales, Australia; 2 Prince of Wales Clinical School, University of New South Wales, Sydney, New South Wales, Australia; 3 Australian Prostate Cancer Research Centre, Institute of Health and Biomedical Innovation, Queensland University of Technology, Brisbane, Queensland, Australia; 4 Institute of Infection Immunology, TWINCORE, Centre for Experimental and Clinical Infection Research, Hannover, Germany; 5 Institut fuer Umweltmedizinische Forschung, University of Düsseldorf, Düsseldorf, Germany; Dana-Farber Cancer Institute, United States of America

## Abstract

Interleukin(IL)-18 is a pleiotrophic cytokine with functions in immune modulation, angiogenesis and bone metabolism. In this study, the potential of IL-18 as an immunotherapy for prostate cancer (PCa) was examined using the murine model of prostate carcinoma, RM1 and a bone metastatic variant RM1(BM)/B4H7-luc. RM1 and RM1(BM)/B4H7-luc cells were stably transfected to express bioactive IL-18. These cells were implanted into syngeneic immunocompetent mice, with or without an IL-18-neutralising antibody (αIL-18, SK113AE4). IL-18 significantly inhibited the growth of both subcutaneous and orthotopic RM1 tumors and the IL-18 neutralizing antibody abrogated the tumor growth-inhibition. In vivo neutralization of interferon-gamma (IFN-γ) completely eliminated the anti-tumor effects of IL-18 confirming an essential role of IFN-γ as a down-stream mediator of the anti-tumor activity of IL-18. Tumors from mice in which IL-18 and/or IFN-γ was neutralized contained significantly fewer CD4^+^ and CD8^+^ T cells than those with functional IL-18. The essential role of adaptive immunity was demonstrated as tumors grew more rapidly in RAG1^−/−^ mice or in mice depleted of CD4^+^ and/or CD8^+^ cells than in normal mice. The tumors in RAG1^−/−^ mice were also significantly smaller when IL-18 was present, indicating that innate immune mechanisms are involved. IL-18 also induced an increase in tumor infiltration of macrophages and neutrophils but not NK cells. In other experiments, direct injection of recombinant IL-18 into established tumors also inhibited tumor growth, which was associated with an increase in intratumoral macrophages, but not T cells. These results suggest that local IL-18 in the tumor environment can significantly potentiate anti-tumor immunity in the prostate and clearly demonstrate that this effect is mediated by innate and adaptive immune mechanisms.

## Introduction

Prostate cancer (PCa) is the second most common cancer of men in the world [1]. Globally in 2002, an estimated 679,000 men were newly diagnosed with PCa and an estimated 221,000 died from it [1]. PCa that metastasizes to bone is virtually incurable [2]. PCa is often considered as an ideal candidate for cancer immunotherapy because of the expression of prostate specific molecules and the non-essential nature of the prostate gland [3]. Experiments using mice have shown that the immune stimulatory effects of some cytokines can induce strong anti-tumor immunity [4]. This involves both innate and adaptive immune mechanisms that function complimentarily to promote tumor immunity [5].

Interleukin (IL)-18 is an 18kd protein that has potential as an anti-cancer agent. It belongs to the IL-1 family of cytokines and is produced by many cell types including Kupffer cells [6], macrophages [7], dendritic cells [8], keratinocytes [9], intestinal epithelial cells [10], osteoblasts [11], astrocytes and microglial cells [12]. The IL-18 receptor (IL-18R) is expressed on macrophages, neutrophils, natural killer (NK) cells and endothelial cells and can be up-regulated on Th1 and B cells by IL-12 [13]. Like IL-1β, pro-IL-18 lacks a signal peptide and is activated into its mature form following cleavage by IL-1β converting enzyme (ICE) [14], [15]. IL-18, originally described as ‘interferon-gamma (IFN-γ) inducing factor’ (IGIF), is a potent inducer of IFN-γ by T cells and natural killer (NK) cells and is synergistic in this function with IL-12 [16]. IL-18 is known to drive the differentiation of CD4^+^ T cells to the Th1 phenotype, favouring cell-mediated immune responses, although it can also promote Th2 responses under some conditions [16]. IL-18 induces the proliferation and enhances the cytotoxicity of both T and NK cells [13]. It activates macrophages to produce IFN-γ and is chemotactic for neutrophils [17], [18]. IFN-γ, a pleiotrophic Th1-promoting cytokine downstream of IL-18, induces expression of MHC class I molecules and Fas (promoting cytotoxic CD8^+^ T cell responses) and up-regulates MHC class II molecules (promoting antigen-specific CD4^+^ T cell responses) [19].

IL-18 has anti-tumor effects in several murine models of cancer. IL-18 gene-transfer into tumor cells, alone [20], [21], [22] or in combination with IL-12 [23], [24], [25] or IL-23 [26], results in tumor growth inhibition. IL-18-mediated tumor stasis is also achieved by administrating the cytokine via intraperitoneal [27], [28], [29], [30], intravenous [27], intratumoral [31] and peritumoral [32] routes. IL-18 as an adjuvant enhances the anti-tumor efficacy of dendritic cell-based vaccines [33], [34], [35] and suicide gene therapies [36]. In those studies, the anti-tumor effects of IL-18 were mediated through enhancement of anti-tumor immunity or inhibition of tumor angiogenesis. Here we investigated the potential of IL-18 as a cancer agent for PCa by using a murine orthotopic model of prostate carcinoma, the RM1 cell line. We provide proof-of-concept that RM1 tumors engineered to secrete bioactive IL-18 exhibit significant growth inhibition of prostate tumors that is mediated by immune potentiation. Whilst in early experiments RM1 tumors were grown subcutaneously, we subsequently generated tumors orthotopically (in the prostate) to better model human disease. Furthermore, direct injection of IL-18 into tumors, a clinically relevant means of administration, also inhibited tumour growth. Furthermore, direct injection of IL-18 into tumors, a clinically relevant means of administration, also inhibited tumor growth.

## Materials and Methods

### Cell lines

RM1 cells were obtained from Dr. Tim Thompson (Baylor College of Medicine, TX, USA) [37]. RM1-IL18, RM1-LACZ and RM1(BM) and derivatives thereof were developed in-house from the RM1 parental cell line. The hybridoma cell line A2, which produces an IgG1 antibody (Ab) against MOPC cancer cells used as a negative control antibody, was provided by Dr Andrew Collins (University of New South Wales, Australia). All these cell lines were maintained in Dulbecco's modified Eagle medium (DMEM) containing 10% Fetal Calf Serum (FCS) and 2 mM L-glutamine (complete DMEM). The hybridomas SK113AE4 (αIL-18) [38] obtained from Prof. Irmgard Förster (Institut für Umweltmedizinische Forschung, Germany) and XMG1.2 (αIFN-γ) were cultured in complete RPMI media (RPMI with 10% Fetal Calf Serum, 2 mM L-glutamine). Cells were incubated at 37°C in a humidified atmosphere of 5% C0_2_/air.

### Ethics Statement

All studies were in accordance with guidelines of the Animal Care and Ethics Committee (ACEC) of the University of New South Wales (ACEC IDs: 06/45A, 06/81B, 08/101B).

### Mice

Male C57BL/6 and C57BL/6 RAG1^−/−^, 4–6 weeks old, were purchased from Laboratory Animal Services, University of Adelaide and Animal Resources Centre, Western Australia, respectively. All mice were maintained at the Biological Resources Centre (BRC), University of New South Wales, Sydney, Australia.

### Generation of RM1-IL18 and RM1(BM)/IL-18^lo^-luc cell lines

The RM1-LACZ cells are plasmid transfected to express the E. coli LacZ gene. The RM1(BM) cell line is a bone-metastatic derivative of RM1 [39] and RM1(BM)/IL18^lo^-luc expresses constitutively active murine IL-18 and firefly luciferase. *RM1-IL18* : RM1 cells were transfected with pVITRO2-GFP/mIL-18 using lipofectamine reagent (Invitrogen, CA, USA) according to the manufacturer's instructions. Briefly, the 3136 bp DNA fragment (which contains the GFP gene) of pVITRO2/GFP/LacZ (In vivogen, CA, USA) was excised with the *Nhe I* and *AvrII* restriction enzymes and inserted into pVITRO2-mcs (In vivogen, CA, USA), replacing the 2452 bp DNA fragment between multiple cloning sites 1 and 2 (now termed ‘pVITRO2-mcs/GFP’). The hybrid mIL-18 gene, containing the signal sequence of IFN-β, excised from the pCEXV3/hybrid mIL-18 plasmid (provided by Dr Isao Hara, Kobe University School of Medicine, Japan) was inserted into the pVITRO2-mcs/GFP plasmid (now termed ‘pVITRO2-GFP/mIL-18’) via the *EcoRI* restriction sites. For transfection, RM1 cells cultured to 80% confluence in 10 cm^2^ wells were incubated with a complex (composed of 1 µg of DNA construct and 10 µl of Lipofetamine 2000) diluted in 2.5 ml of serum free Opti-Mem (Invitrogen, CA, USA). Selection for transfectants began 72 hours post transfection by culture in complete DMEM containing hygromycin b (Invitrogen, CA, USA) at 400 µg/ml and maintained at 200 µg/ml once stable populations were generated (RM1-IL18 cells). A control cell line (RM1-LACZ) in which the plasmid contained the gene for LACZ in place of mIL-18, was generated in a similar way. *RM1(BM)/IL-18^lo^-luc*: The RM1(BM) cell line was transfected with plasmid phCMV-CLUC containing the gene for firefly luciferase under control of the human CMV promoter (Genlantis, CA, USA) and a luciferase expressing clone was isolated which was named RM1(BM)/B4H7-luc (Power et al., unpublished). RM1(BM)/B4H7-luc cells were subsequently transfected with pVITRO2-blasti/mIL-18. Briefly, the 605 bp DNA fragment containing the hybrid IL-18 gene was excised from pCEXV3/hybrid mIL-18 plasmid and inserted into pVITRO2-blasti/mcs (In vivogen, CA, USA) at the *BglII* and *XhoI* restriction sites. The protocol for transfection was similar to that of RM1-IL18 except that selection involved blasticidin at 10 µg/ml (In vivogen, CA, USA).

### Determination of IL-18 expression by RM1-IL18 and RM1(BM)/IL18^lo^-luc

Conditioned media were prepared by culturing tumor cells to 80% confluence (in T75 flasks) followed by 48 hour incubation in 10 ml of serum-free DMEM. IL-18 was detected by sandwich ELISA assay using the Pharmingen OPTEIA mouse anti-IL-18 antibody set (BD Biosciences, CA, USA). The concentration of IL-18 in conditioned media of RM1-IL18 cells was interpolated from results obtained for purified recombinant IL-18 (Medical & Biological Laboratories, Japan) at concentrations ranging from 0 ng/ml to 100 ng/ml; limit of detection of the ELISA: 12 ng/ml.

### Purification of monoclonal antibodies for *in vivo* studies

The hybridomas A2 (mouse IgG1), SK113AE4 cells (αIL-18; mouse IgG1) and XMG1.2 (αIFN-γ; rat IgG1) were cultured as described until the media were exhausted, and the secreted antibody was harvested. The hydridoma culture supernates were passed through a protein G sepharose column (Amersham Biosciences, NJ, USA). The bound IgG was eluted using 0.1 M glycine, pH 3, and the pH was immediately neutralized with 1 M Tris-base, pH 8.0. The eluates were dialysed against PBS and the purity of antibody preparations was determined by SDS-PAGE followed by Coomassie blue (Biorad, CA, USA) staining. The concentration was determined by BCA protein assay (Pierce Biotechnology, IL, USA).

### Tumorigenesis studies

Cultured RM1-IL18, RM1-LACZ, RM1(BM)/B4H7-luc and RM1(BM)/IL-18^lo^-luc cells were lifted from culture flasks with trypsin in PBS, washed twice with PBS and resuspended in PBS. Cell counts were performed with a hemocytometer and viability was assessed by trypan blue exclusion. All animal experiments involved implantation with either 6×10^5^ or 2×10^6^ tumor cells in mice (see figure legends for individual experiments). Subcutaneous implantation of cells was performed on the right shoulder or flank of mice in 100 µL volume. These tumors were measured using electronic calipers every 2-3 days and tumor volume calculated from the formula for the volume of an elipse: V  =  π/6(d_1_.d_2_)^3/2^, where d_1_ and d_2_ are two perpendicular tumor dimensions [40]. For intraprostatic implantation, the hair was removed from the abdomen of mice using a depilatory and the mice were anesthetised with isoflurane inhalation anesthetic. A low abdominal transverse incision was made through the skin and abdominal muscle and the bladder was grasped with forceps and pulled through the incision, exposing the prostate. Tumor cells were injected into the ventral prostate in 50 µl volume. The bladder was returned to the abdomen and the incision closed with sutures. *In vivo* cytokine neutralization was accomplished by administering 500 µg of neutralizing αIL-18 antibody and/or αIFN-γ antibody, or A2 control antibody into the tail vein or intraperitoneally 30 minutes prior to tumor-cell implantation. Each experimental group consisted of 5–10 mice. All mice were weighed and checked for signs of distress regularly. Abdominal palpation was used to monitor tumor size. For intratumoral injection of IL-18, RM1(BM)/B4H7-luc cells were implanted on the right flank of mice. 5 daily injections of 1.5 µg of IL-18 in 15 µl volume commenced when the tumors reached 6 mm × 6 mm in dimension. Control mice were injected with vehicle only.

### Depletion of immune cell subsets in tumor studies

For depletion of CD4^+^ and CD8^+^ T cells, mice were injected intraperitoneally with 100 µg of MAbs GK1.5 or 2.43 (both from Bio X Cell, NH, USA), respectively, four days and two days prior to tumor-cell implantation and subsequently every 4 days thereafter until day 18. For depletion of NK cells, mice were treated with 20 µl of anti-asialo GM1 anti-serum (Wako Pure Chemicals, TX, USA) four days and one day prior to tumor-cell implantation, and every 5 days thereafter. Depletion of >95% of each immune cell subset was confirmed by flow cytometry performed on peripheral blood on day 7, and again on spleen cells following necropsy on day 17.

### Tumor bioluminescence imaging

Bioluminescent imaging was performed using a Xenogen IVIS Lumina (Xenogen, CA, USA). For *in vitro* imaging, bioluminescent RM1(BM)/IL-18^lo^-luc cells were seeded at 50,000 cells/well down to 50 cells/well (2-fold serial dilution) in 96-well plates. D-luciferin (Xenogen, CA, USA) was added to each well (final concentration was 150 µg/ml of media) 3–5 mins prior to imaging. For *in vivo* imaging, mice were injected ip with D-luciferin diluted in PBS (15 mg/ml stock) at 150 mg/kg. Mice were anaesthetised and imaged 8–12 minutes after injection with D-luciferin. Bioluminescence was analysed using Living Imagine software (Xenogen, CA, USA). For *in vivo* imaging, the total flux in photons/second (p/s) within each defined region of interest provides a surrogate of tumor size.

### Flow cytometric analysis

The cellular profile of tumor draining lymph nodes was analysed by flow cytometry using the FACScan (Becton Dickinson, CA, USA). Para-aortic lymph nodes were disintegrated by compression between the frosted sides of 2 microscope slides, and suspended in PBS containing 5% turbo calf serum. Removal of tissue clumps and debris was achieved by layering the suspension over 2 ml of cold FCS, followed by collection of the cells which remain suspended above the serum. T cells were stained with αCD3-FITC antibody (17A2), αCD4-PE (H129.19) and biotinylated-αCD8a antibody (53-6.7) plus PerCP-streptavidin (BD Biosciences Pharmingen, CA, USA). NK cells were detected using αNK1.1-PE (PK136) and αCD3-FITC antibodies. B cells were identified by incubation with αI-A^b^ (MHC-II)-FITC antibody (AF6-120.1) and biotinylated-αCD19 (1D3) plus PerCP-streptavidin. All antibodies were from BD Biosciences Pharmingen, CA, USA. These antibodies do not cross react with the cell-depleting antibodies.

### Measurement of cytokines in mice serum

The concentration of IFN-γ, IL-12, GM-CSF, MCP-1, RANTES and IL-4 in serum collected at necropsy was determined using BD Cytometric Bead Array technology (BD Biosciences, CA, USA), according to the manufacturer's instructions. Briefly, the microbeads, which have distinct fluorescence intensities and are coated with capture antibodies for each cytokine, were incubated with recombinant standard or serum (diluted 1∶6) for 1 hour. PE-conjugated detection antibodies were then added and incubated for a further 1 hour. The beads were washed and resuspended in wash buffer and then analysed using a BD FACS Canto (BD Biosciences Pharmingen, CA, USA). Concentration of cytokine level was determined using CBA analysis software.

### Histochemical and immunohistochemical staining

For histological examination, resected tumors were fixed in zinc-fixative (BD Biosciences Pharmingen, CA, USA) and embedded in paraffin for sectioning. Sections of 5 µm thickness were processed using standard protocols. Haematoxylin & Eosin (H&E) staining was carried out to assess tissue morphology and for assessment of cellular infiltration. Immunohistochemical staining was performed using primary antibodies to CD8a (53-6.7), CD4 (H129.19) (all from BD Biosciences Pharmingen, CA, USA), asialo-GM1 (Wako Pure Chemicals, TX, USA), F4/80 (BM8) (eBioscience, San Diego, CA, USA), caspase-3 (Cell Signaling Technology, MA, USA), Ki-67 (TEC-3) (DAKO, Glostrup, Denmark) and IL-18 (Santa Cruz, CA, USA). The secondary antibodies used were biotinylated rabbit α-rat, biotinylated goat α-rabbit or biotinylated rabbit α-goat (all from Vector Laboratories, CA, USA). Histochemical detection was achieved using Biotin-Avidin Complex (Vector, CA, USA) followed by incubation with 3,3′ Diaminobenzidine (DAB) substrate (DAKO, Glostrup, Denmark).

### Assessment of tumor-infiltrating immune cells

Histological assessment was performed using an Olympus BX51 microscope. Tumor sections stained for CD8 and CD4 were examined by microscopy at 200× magnification for assessment of numbers of tumor-infiltrating lymphocytes. Entire sections were progressively scanned and the number of lymphocytes in each field of view was recorded in a semi-quantitative fashion as either zero, between one and ten, between eleven and fifty or greater than fifty. This approach not only provides an indication of the number of lymphocytes infiltrating the tumor, but also their relative distribution within the tumor. Tumors stained for F4/80, Asialo GM1, Caspase-3, Ki67 and H&E were also scanned at 200× magnification and were qualitatively assessed as described in Results.

### Statistical Analysis

All statistical tests were performed using GraphPad Prism software. Appropriate statistical tests were employed depending on the nature of the data being analysed. Refer to the figure legends for specific tests used for data from individual experiments.

## Results

### Confirmation of IL-18 expression by RM1-IL18 cells

IL-18 was detected in the conditioned media of RM1-IL18 cells and RM1(BM)/IL18^lo^-luc cells by ELISA (10^6^ cells released >200 ng and ∼20 ng respectively in 48 hours). IL-18 expression was also confirmed by western blot (data not shown) prior to each experiment. IL-18 was not expressed by RM1 parental, RM1-LACZ or RM1(BM)/B4H7-luc cells. The biological activity of the secreted IL-18 was confirmed by its ability to induce IFN-γ production from murine splenic T cells (Figure S1 A). IFN-γ production was induced in a dose dependent manner following incubation with varying concentrations of RM1-IL18 conditioned media. Immunohistochemically, RM1-IL18 tumors grown in the prostate were positive for IL-18, confirming its secretion *in vivo* (Figure S1 B).

### IL-18 inhibits the growth of subcutaneous and orthotopic prostate carcinomas

**Figure 1:**
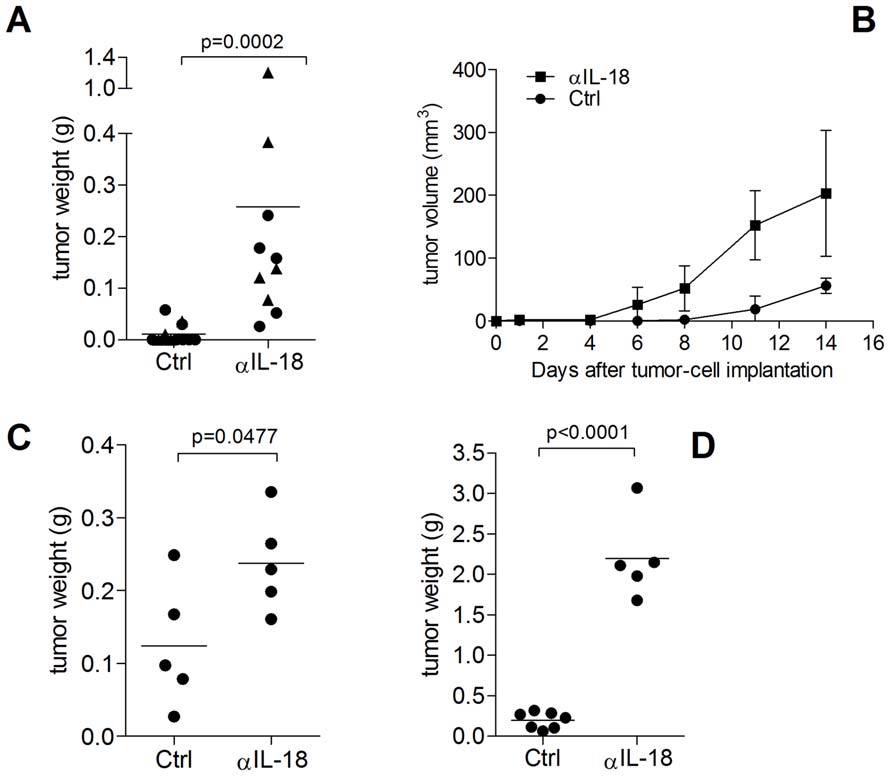
IL-18 inhibits RM1 tumor growth. IL-18 expression by RM1 murine prostate carcinoma cells inhibits the *in vivo* growth of these cells implanted subcutaneously and orthotopically into syngeneic mice, but not when IL-18 is inactivated by the IL-18-neutralizing antibody (αIL-18), SK113AE4. A) Post mortem weights of RM1-IL18 tumors from mice treated with (αIL-18) or without (Ctrl) the IL-18-neutralizing antibody after subcutaneous implantation of 6×10^5^ tumor cells. Results in (A) are from two independent experiments (circles and triangles) in which mice were killed on day 14. B) Growth rates of subcutaneous RM1-IL18 tumors in mice with or without IL-18-neutralization are shown. C) Post mortem weights of control RM1-LACZ tumors (which do not express IL-18) from mice treated with αIL-18 or without (Ctrl), 11 days following subcutaneous injection of 6×10^5^ cells. Post mortem weights of RM1-IL18 tumors 12 days after 6×10^5^ cells were implanted orthotopically, with or without IL-18 neutralization are shown in (D). Statistical analysis performed for (A) was the Mann-Whitney test, and (c) and (d) was the unpaired T-test. Significant p values are shown where appropriate.

To investigate the effects of local secretion of IL-18 by tumors on tumor growth *in vivo*, RM1-IL18 cells were implanted subcutaneously or intraprostatically into syngeneic C57BL/6 mice with or without the IL-18-neutralizing antibody (αIL-18), SK113AE4. 100% of mice that were injected with RM1-IL18 cells subcutaneously and received the αIL-18 Ab treatment developed tumors (10 of 10); in contrast only 33% of mice that did not receive the neutralizing antibody (Ctrl group) developed tumors (4 of 12) (Fig. 1 A). The subcutaneous tumors that developed in the latter group showed delayed onset and were significantly smaller (p = 0.0002) at necropsy compared to those given αIL-18 (Fig. 1 B), indicating that IL-18 not only prevented tumor formation but also suppressed the growth of those that developed. In mice implanted subcutaneously with the control cell line, RM1-LACZ, IL-18 neutralization resulted in significantly larger tumors (p = 0.0477) (Fig. 1 C), suggesting that endogenous IL-18 plays a role in tumor immunity. All mice implanted with RM1-IL18 tumor cells orthotopically (in the prostate) developed tumors (Fig. 1 D), however, neutralization of IL-18 through αIL-18 treatment resulted in significantly larger tumors (p<0.001).

### Anti-tumor effects of IL-18 require IFN-γ

**Figure 2:**
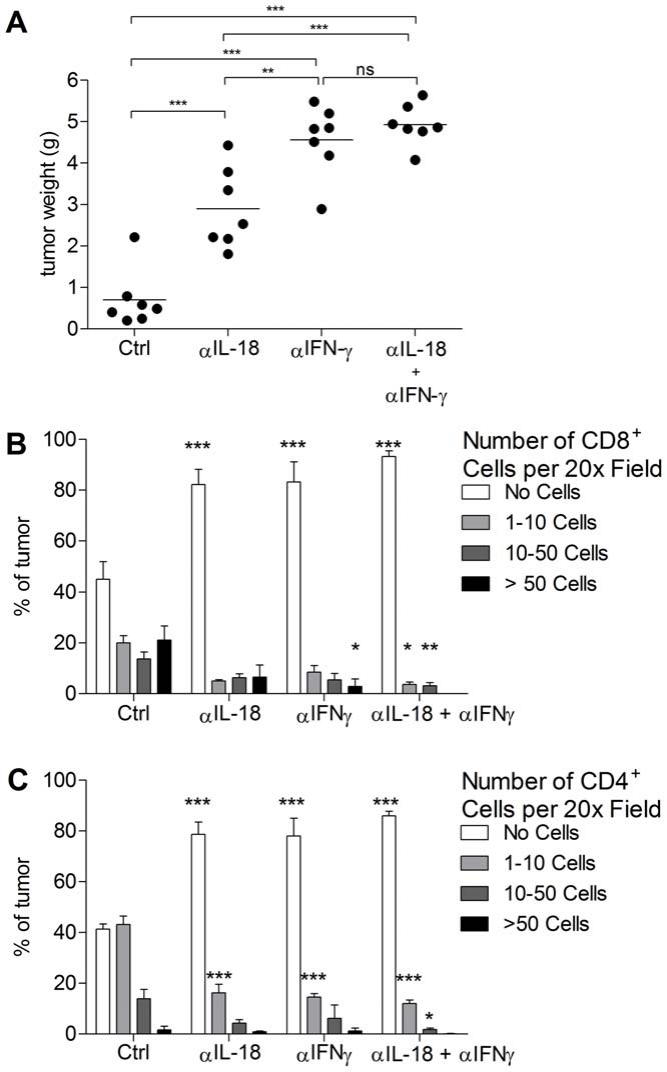
The anti-tumor effects of IL-18 are mediated predominantly through IFN-γ. 6×10^5^ RM1-IL18 cells were implanted orthotopically in syngeneic, immunologically intact C57BL/6 mice. A) Post mortem tumor weights, 14 days after cell injection are shown. Mice were treated with IL-18-neutralizing antibody (αIL-18), and/or IFN-γ neutralizing antibody (αIFN-γ) and/or A2 isotype control antibody (Ctrl), as indicated. Statistical analysis was performed by one-way ANOVA followed by Tukey's multiple comparison test. B) & C) Semi-quantitative assessment of CD8^+^ and CD4^+^ T cells, in tumors from mice in (A). Histological sections were immunohistochemically stained for CD8 (B) and CD4 (C) and the number of each T cell type per microscopic field at 200× magnification was determined as either zero, 1–10, 11–50, or >50 as described in Methods and Materials. Statistical analysis was performed by two-way ANOVA followed by Bonferroni post test. * p value 0.01 to 0.05, ** p value 0.001 to 0.01, *** p<0.001.

Since IFN-γ is an effector molecule downstream of IL-18 with important functions in Th1-responses, we hypothesized it may mediate the anti-tumor effects of IL-18. To investigate this, RM1-IL18 cells were implanted orthotopically in C57BL/6 mice and treated with neutralizing antibodies to IL-18, IFN-γ or both. Neutralization of IL-18 and/or IFN-γ resulted in significantly larger tumors compared to those given the control antibody (Ctrl) (Fig. 2 A). As previously, tumors in mice treated with the IL-18-neutralizing antibody alone were larger than controls (Ctrl group). Neutralization of IFN-γ alone resulted in larger tumors than those in Ctrl mice or αIL-18 treated mice (Fig. 2 A). Co-neutralization of IL-18 and IFN-γ did not result in tumors larger than those given IFN-γ antibody alone.

### IL-18 correlated with significant tumor infiltration of neutrophils, macrophages, CD8^+^ T cells and CD4^+^ T cells but not NK cells

**Figure 3:**
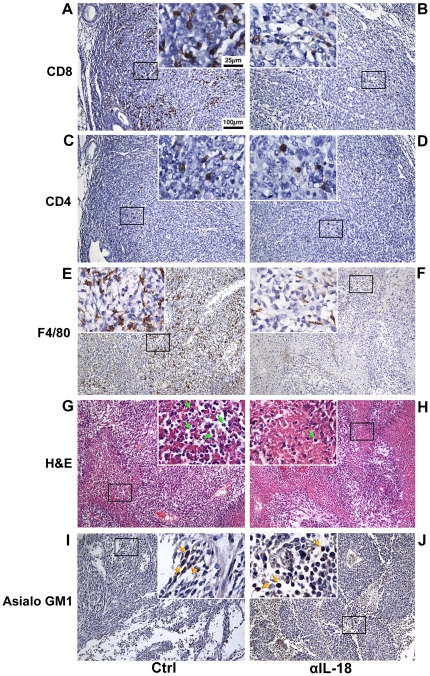
Immune cell infiltration of orthotopic RM1-IL18 tumors. Histochemical and immunohistochemical staining of intraprostatic RM1-IL18 tumors from C57BL/6 mice described in the legend to Fig. 2 A, without (A, C, E, G & I) or with (B, D, F, H & J) αIL-18 treatment. Immunohistochemical staining was performed with anti-CD8 (A & B), anti-CD4 (C & D), anti-F4/80 for macrophages (E & F) and anti-asialo-GM1 for NK cells (I & J). H&E staining of tumor sections shows neutrophil infiltration (G & H), indicated by green arrowheads.

In RM1-IL18 tumors (from Fig. 2 A), IL-18 correlated with significantly greater tumor infiltration of CD8^+^ (Fig. 2 B) and CD4^+^ cells (Fig. 2 C). In the Ctrl group, 18.8% percent of the tumor contained >50 CD8^+^ cells per microscopic field compared to 6.6%, 2.9% and 0.0% respectively for groups that received neutralizing antibodies to IL-18, IFN-γ, or both (Fig. 2 B, Fig. 3 A & B). Furthermore, 13.9% of the tumor in the Ctrl group contained between 11 and 50 CD4^+^ cells per microscopic field compared to 4.3%, 6.2% and 1.8% respectively for the mice in which IL-18, IFN-γ, or both were neutralized (Fig. 2 C and Fig. 3 C & D). Profound tumor-infiltration of macrophages was observed in the Ctrl treatment group, but less so when IL-18 was inactivated (Fig. 3 E & F). H&E staining revealed that mice in which the IL-18 was not neutralized had greater numbers of neutrophils within tumors, compared to those from mice given αIL-18 (Fig. 3 G & H). No correlation between IL-18 and numbers of NK cells was found in normal C57BL/6 mice (Fig. 3 I & J).

We also examined the degree of apoptosis and cell proliferation in RM1-IL18 tumors in normal mice from Fig. 2. Tumors from mice given the Ctrl antibody had more staining for the apoptotic marker, caspase-3, compared to those from mice given αIL-18, αIFN-γ, or both (Figure S2 A & B, E). An inverse correlation was observed with Ki-67 staining (Figure S2 C & D, E).

### Innate and adaptive immune mechanisms mediate the anti-tumor effects of IL-18

**Figure 4:**
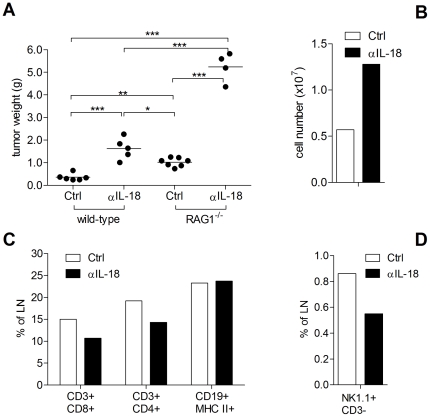
Both innate and adaptive immune mechanisms are involved in inhibition of tumor growth by IL-18. 6×10^5^ RM1-IL18 cells were implanted orthotopically in syngeneic, immunologically intact (wild-type) C57BL/6 mice or immunodeficient C57BL/6 RAG-1^−/−^ mice (one single experiment). A) Post mortem tumor weights 14 days after cell injection is shown. Mice were treated with (αIL-18) or without (Ctrl) the IL-18-neutralizing antibody. Statistical analysis was performed by one-way ANOVA followed by Newman-Keuls multiple comparison test. Significant p values are shown. * p value 0.01 to 0.05, ** p value 0.001 to 0.01, *** p<0.001 B) Tumor-draining lymph nodes of normal C57BL/6 mice with orthotopic RM1-IL18 tumors show increased cellularity but a decrease in lymphocytes as a percentage of total cells (C & D) when IL-18 is neutralized as assessed by flow-cytometry. Results shown are for pooled para-aortic lymph nodes from normal C57BL/6 mice described in Figure 4 A.

RM1-IL18 tumors grown in the prostate of RAG1^−/−^ mice, which lack T and B cells, were significantly larger than those from their corresponding treatment group in normal C57BL/6 mice (Fig. 4 A). This demonstrates an important role for adaptive immunity in mediating the anti-tumor effects of IL-18. In both mouse strains, αIL-18 treatment resulted in significantly larger tumors than in corresponding Ctrl mice, indicating that apart from adaptive immunity, other mechanisms including innate immune mechanisms are involved in this tumor inhibitory effect. Flow cytometric analysis from lymph nodes of normal tumor-bearing C57BL/6 mice showed that αIL-18 treatment was associated with greater cellularity in the tumor-draining lymph node (Fig. 4 B). However, as a percentage of total cells, CD8^+^ T cells, CD4^+^ T cells (Fig. 4 C) and NK cells (Fig. 4 D) were higher when IL-18 was present.

RM1-IL18 tumors generated in immunocompromised RAG1^−/−^ mice (from Fig. 4 A) from the Ctrl treatment group had greater infiltration of macrophages and neutrophils compared to IL-18-neutralized tumors (Figure S3 A–D) showing tumor infiltration of macrophages and neutrophils in RAG1^−/−^ mice). However, the abundance of macrophages in tumors of RAG1^−/−^ mice was less marked than in normal C57BL/6 mice. Noteworthy is that neutrophils appeared to localize within the apoptotic/necrotic regions of the tumor whereas the macrophages infiltrated the viable areas. Similar to normal mice, no correlation between IL-18 and numbers of NK cells was found in RAG1^−/−^ mice (Figure S3 E & F).

### CD4^+^ and CD8^+^ T cells, and NK cells are involved in the anti-tumor effect of IL-18

**Figure 5:**
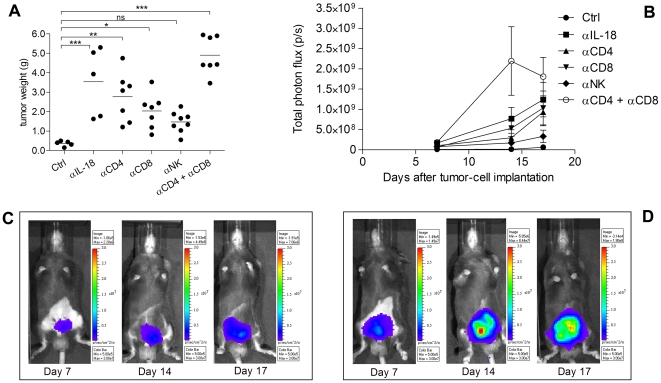
Anti-tumor effects of IL-18 are mediated by CD4^+^ and CD8^+^ T cells, and NK cells. C57BL/6 mice were implanted with 2×10^6^ RM1(BM)/IL-18^lo^-luc cells orthotopically, and treated with 500 µg of either the αIL-18 antibody or the A2 isotype control (Ctrl) antibody. Some mice were treated with αCD4 (GK1.5), αCD8 (2.43) or αNK (Asialo GM1), as indicated (See Materials and Methods). All mice depleted of any cell subset also received the A2 isotype control antibody. A) post-mortem tumor weights on day 17. Statistical analysis was performed by one-way ANOVA followed by Dunnett's multiple comparison's test to the corresponding Ctrl. * p value 0.01 to 0.05, ** p value 0.001 to 0.01, *** p<0.001. B) Average tumor bioluminescence in each treatment group at three time points. Representative images of tumor bioluminescence in mice treated with Ctrl (C) or αIL-18 antibodies (D).

Bioluminescent RM1(BM)/IL-18^lo^-luc tumors (Figure S1 C), which confirms that RM1(BM)/IL-18^lo^-luc cells are bioluminescent *in vitro* prior to implant) generated orthotopically were significantly larger in mice depleted of CD4^+^ and/or CD8^+^ T cells, compared to undepleted mice (Ctrl) (Fig. 5 A). Depletion of NK cells also resulted in larger tumors compared to the Ctrl group, although this effect not statistically significant (Fig. 5 A). Neutralisation of IL-18 resulted in larger tumors compared to Ctrl treatment, confirming previous observations (Fig. 5 A). Tumors from mice depleted of immune cell subsets had faster growth rates throughout the experiment as assessed by bioluminescent imaging (Fig. 5B–D ).

### Intratumoral injection of IL-18 into pre-formed subcutaneous tumors inhibits their growth

**Figure 6:**
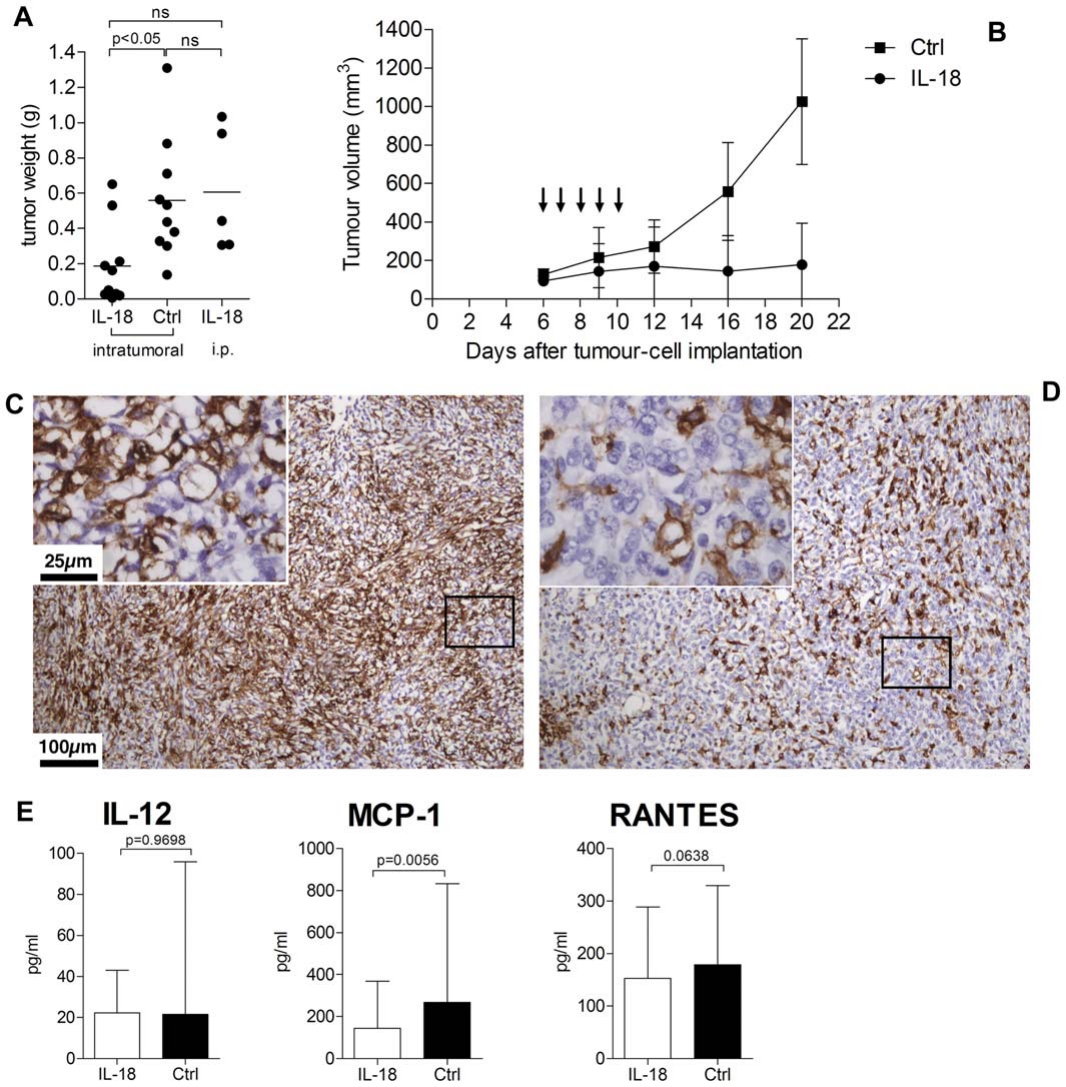
Intratumoral injection of rIL-18 inhibits growth of subcutaneous tumors. C57BL/6 mice were injected subcutaneously with 2×10^6^ RM1(BM)/B4H7-luc cells. IL-18 (1.5 ug) or vehicle (Ctrl) was injected intratumorally in 15 µl volume, or 100 µl when injected i.p., for 5 consecutive once tumors reached 6 mm ×6 mm in size. A) post-mortem tumor weights from two independent experiments which showed similar results. Statistical analysis was performed by non-parametric Kruskal-Wallis test followed by Dunn's multiple comparison's test. B) Growth rates of subcutaneous tumors injected intratumorally with IL-18 or vehicle (Ctrl) are shown from one representative experiment. Staining for F4/80 (macrophages) in tumors treated with IL-18 (C) or vehicle (D) is shown. E) Concentration of cytokines in mice serum from Fig. 6 A is shown. Graphs show median concentration and range. Statistical analysis was performed by non-parametric Mann-Whitney test.

Direct injection of 1.5 µg of recombinant IL-18 into pre-established subcutaneous RM1(BM)/B4H7-luc tumors for 5 consecutive days inhibited their growth (Fig. 6 A). Vehicle injection had no effect on tumor growth as all tumors progressed (Fig. 6 B). IL-18 treatment resulted in complete tumor regression in 3 of 10 mice (Fig. 6 A). Once the IL-18 treatment was started, the tumors either regressed or remained relatively stable through to day 20 (Fig. 6 B). In contrast intraperitoneal injection of IL-18 had no effect on tumor growth (Fig. 6 A). Immunohistochemistry of tumors injected with IL-18 showed greater infiltration of macrophages compared to control groups (Fig. 6 C & D). No difference in the numbers of CD4^+^ T cells, CD8^+^ T cells or NK cells were seen between the two groups (data not shown). IFN-γ and GM-CSF and IL-4 were not present at detectable levels in the serum of these mice and there was no difference in IL-12 or RANTES between treatments. However, αIL-18 treatment correlated with higher levels of MCP-1 (Fig. 6 E).

## Discussion

This study demonstrates that IL-18 in the RM1 tumor environment, achieved either by IL-18 gene-introduction or direct injection can slow the growth of both subcutaneous and orthotopic tumors in *in vivo* models, thus provides proof of concept that IL-18 has potential as an immunotherapeutic agent for prostate cancer. IL-18 either prevented the establishment or at least slowed the growth of subcutaneous tumors, but perhaps more importantly, slowed the growth of orthotopic tumors. The difference in growth patterns at the two anatomical sites highlights the importance of using orthotopic models to assess not only the effect of immunotherapy on tumour growth but also the mechanisms involved in mediating such effects. The anti-tumor effect was dependent on IFN-γ as inactivation of this cytokine rendered IL-18 incapable of inducing tumor stasis. Adaptive immune mechanisms were demonstrated to be important in mediating the anti-tumor effects of IL-18, and innate mechanisms are also implicated.

Generation of intraprostatic RM1 tumors in syngeneic mice is a useful model of prostate cancer in humans and allows assessment of the anti-tumor immune response in this organ rather than assuming that the results will be similar to those found with heterotropic implantation sites such as sc. We know that tumor take rates and growth kinetics will vary depending on the anatomical site of implantation due to factors which are likely to include the nature of the stroma in which the cells are transplanted, the local vasculature, the prevalence of locally-produced tumor growth factors, the anti-tumor immune response at the site of implantation, and in studies where therapeutic interventions are assessed, the local bio-availability of the compound being tested. Thus orthotopic models are preferable for modeling human disease. Although RM1 tumors grew subcutaneously, orthotopic implantation of these tumor cells proved more conducive for tumor growth (Fig. 1 A-D). Our results also suggested that there are significant differences in the nature of the immune response to tumors at the different anatomical sites, a finding which is not unexpected, and we are exploring these differences further. Because of this, we employed intraprostatic implantation of RM1 cells in further experiments to investigate the anti-tumor mechanisms induced by IL-18 in the prostate. Furthermore, whilst several reports compare the *in vivo* growth characteristics of derivative cell lines transfected with different constructs to demonstrate the tumor suppressive effects of IL-18 [20], [22], [23], [25], in our studies the use of an IL-18 neutralizing antibody confirms that any observed difference in tumor growth can be attributed to the cytokine and is not due to inherent differences between cell lines.

In all our experiments, regardless of the site of injection of the tumor cells or method of delivery, IL-18 reduced the tumor burden significantly. Of interest also is the observation that neutralization of endogenous IL-18 allows tumors to grow more rapidly indicating that in this model IL-18 is important for tumor immune surveillance (Fig. 1 C). In line with this observation, single nucleotide polymorphisms (SNPs) within the human IL-18 gene that are associated with lower promoter activity and lower gene expression [41], may influence susceptibility to certain cancers including prostate cancer [42], nasopharyngeal cancer [43] and esophageal squamous cell carcinoma [44]. Moreover, human prostate cancer cell lines and clinical PCa specimens are reported to express IL-18 receptor α [45] and IL-18 expression was associated with better patient outcome. However, systemic administration of recombinant human IL-18 into cancer patients, as reported in three clinical trials, did not lead to overt anti-tumor efficacy [46], [47], [48]. Based on our work, we speculate that this route of administration of IL-18 does not deliver therapeutic doses of the cytokine to the tumor to induce anti-tumor responses. Similar to these results, systemic administration of IL-18 could not hinder the growth of subcutaneous tumors (Fig. 6 A). IL-18 only inhibited tumor growth when administered directly into the tumor mass (intralesional) (Fig. 6 A) or if the cytokine was secreted by tumor cells. This indicates that IL-18 is far more effective at the tumor site in generating anti-tumor effects. Intratumoral injection of immune-modulatory compounds is a potentially feasible delivery approach, with administration of recombinant IL-12 (for head and neck squamous cell carcinoma (H&NSCC) [49] and viral vectors encoding cytokine genes IFN-γ [50], GM-CSF [51] (both for melanoma) and IL-12 [52] (colorectal and pancreatic cancers) already being evaluated in clinical trials. In previous studies, numerous mechanisms have been shown to be involved in mediating the anti-tumor effects of IL-18. These include the recruitment and activation of various innate and adaptive immune cells and inhibition of angiogenesis. We explored the involvement of a number of these mechanisms in our studies.

IL-18 is a potent inducer of IFN-γ production by T cells, NK cells and macrophages and is synergistic in this function with IL-12 [16]. The results presented here clearly demonstrate that for intraprostatic tumors the anti-tumor effects of IL-18 are primarily mediated through IFN-γ because neutralization of IFN-γ completely abolished IL-18-mediated protection (Fig. 2 A). This finding is consistent with that of Nagai et al. where IFN-γ was required for the tumor inhibitory effects of IL-18 on B16 melanomas using a gene-transfer approach, although the main mechanism reported in this case was via inhibition of tumor angiogenesis [20]. Osaki et al., however, showed that the tumor suppressive effects of IL-18 on CL-8 tumors are not completely impaired in IFN-γ gene KO mice [30].

Tumors grew more rapidly in RAG1^−/−^ mice, which lack T and B cells, than in normal immunologically intact mice (Fig. 4 A) and together with the depletion results presented in Fig. 5 A & B, indicate that adaptive immunity is involved in the control of tumor growth. Furthermore, in immunologically intact mice, the presence of IL-18 induced significantly greater infiltrations of CD8^+^ T cells and CD4^+^ T cells (Fig. 2 B & C, Fig. 3 A–D) further supporting a role for these cells. In addition, although the tumor draining lymph nodes of IL-18-treated mice had fewer CD4^+^ T cells, CD8^+^ T cells and NK cells per lymph node, they represented a higher percentage of lymph node cellularity than those of the αIL-18 group (Fig. 4 B–D). Although CD4^+^ and CD8^+^ cells represent a smaller percentage of the total cells in the lymph nodes in mice treated with anti-IL18, the absolute numbers of these cells in the draining lymph nodes is higher in the anti-IL18 treated mice. This is not surprising given that these measurements were done following necropsy of mice at the end of the experiment. At this late stage of the experiment the tumors in the treated mice are significantly larger than in the untreated mice and concentration of the neutralizing antibody available will be significantly reduced due to normal metabolic processes (half life of IgG1 is ∼21 days) and consumption. Thus at the end of the experiment, even though there appears to be a significant immune response to the tumour, it is ineffective against a large established tumor. Similar findings have been described previously in concomitant immunity studies [53], [54]. The importance of these cells in the anti-tumor effect are not surprising given that IL-18 is chemoattractive for T cells [55], drives the differentiation of CD4^+^ T cells to the Th1 phenotype, induces the proliferation and enhances the cytotoxicity of T cells and NK cells [16] and induces the maturation of dendritic cells [56].

Our results also suggest a role for innate immunity in IL-18-induced tumor suppression. In RAG1^−/−^ mice, which fail to develop adaptive immunity, the presence of IL-18 resulted in significantly smaller tumors (Fig. 4 A). Furthermore, in both RAG1^−/−^ and immunologically intact mice, IL-18 correlated with greater tumor infiltration of macrophages (Fig. 3 E & F, Figure S3 A & B) and neutrophils (Fig. 3 G & H, Figure S3 C & D). An involvement of macrophages in the anti-tumour effect of IL-18 is further supported by the finding that intratumoral injection of rIL-18 into subcutaneous tumours elevated serum levels of MCP-1 (Fig. 6 E). Macrophages express the IL-18R, and can be activated by and secrete IFN-γ [17], and may be central to the development of adaptive immunity induced by IL-18. Kito et al. demonstrated that macrophages activated through stimulation with IL-12 and IL-18, produce IFN-γ which induces nitric oxide secretion that is cytotoxic for tumors [17]. IL-18 is chemoattractive for neutrophils [18], induces the expression of IL-8 which is chemotactic for neutrophils [57] and under proinflammatory conditions promotes neutrophils to secrete cytotoxic molecules [58]. Interestingly, macrophages appeared to localize in the viable regions (Ki-67+) of the tumors, whilst neutrophils were almost exclusively found in apoptotic areas (caspase-3+) (Figure S2 A–E). NK cells may play a role as depletion of this cell type appeared to inhibit the anti-tumor effect of IL-18, although the difference was not statistically significant in these studies (Fig. 5 A). There was no difference in the number of NK cell infiltration in tumors, in the presence or absence of IL-18 (Fig. 3 I & J, Figure S3 E & F), but may be functionally enhanced by IL-18 as they express the IL-18 receptor [13].

The reported mechanisms of action of IL-18-induced anti-tumor immunity in previous studies have varied. Osaki et al. showed that intraperitoneal injection of recombinant IL-18 for 7 consecutive days either prior to, or after intradermal implantation of CL-8 melanoma cells suppressed tumor growth [30]. This anti-tumor effect was dependent on NK cells, although the importance of T cells was not defined [30]. In contrast to our data, IL-18 treatment did not change tumor infiltration of CD4^+^ T cells or macrophages, and reduced the number of CD8^+^ T cells. Several differences in the experimental models can account for the differences in results. Our RM1 tumors constitutively express IL-18 locally in the tumor resulting in the observed influx of CD4^+^ T cells, CD8^+^ T cells and macrophages into the tumors, while the Osaki et al. model used intraperitoneal injection of IL-18 which is less likely to induce local tumor effects but more likely to induce systemic effects, in this case NK cell activation. Secondly, Osaki et al. reported a reduction in tumor-infiltrating CD8^+^ T cells in tumors that were excised at day 6 of tumor growth [30], a point at which the adaptive immune response is still developing and innate responses are likely to dominate. In contrast, our studies extended 14 days after tumor implantation and thus the adaptive immune response is likely to dominate. Differences in the local tumor microenvironment (intradermal vs intraprostatic and subcutaneous) and the inherent immunogenecity of the tumor cells will also influence the effects of the cytokine.

Yoshimura et al. [22] showed that IL-18-gene transfected CT26 tumors grew more rapidly when CD4^+^ T cells and CD8^+^ T cells were depleted, and tumors from non-depleted mice contained large numbers of infiltrating CD8^+^ T cells, results comparable to our data. In that study, depletion of NK cells did not affect the tumor growth [22]. However, Micallef et al. showed that NK cells are required for IL-18-mediated control of intraperitoneal Meth A sarcoma cells when IL-18 is delivered ip 3 days and 6 hours prior to tumor cell injection [27]. Nagai et al., showed that neither CD8^+^ T cells nor NK cells were involved in the inhibition of IL-18-gene-transfected B16 melanomas as depletion of these cell types had no effect on tumor growth, but growth inhibition was mediated through IL-18-mediated angiostasis [20].

Although both approaches in delivering IL-18 inhibited tumor growth, the mechanisms of action are seemingly different. Intratumoral injection of IL-18 resulted in significant infiltration of macrophages but not T cells within the tumor mass. The lack of correlation between IL-18 and T cell numbers in these tumors contrasts with the situation of IL-18-secreting tumors. This difference is likely to be attributed to the varied route of administration of IL-18: daily injections into pre-established tumors for 5 consecutive days starting from day 6, versus IL-18 expression by tumors throughout. Hence, the kinetics, duration and the magnitude of immune stimulation are different. Other studies have also shown that intratumoral injection of IL-18 can inhibit tumor growth. Kikuchi et al. showed that intratumoral but not intraperitoneal injection of rIL-18 into murine SR-B10A gliomas in the frontal lobe of mice significantly prolonged their survival. This effect was mediated by NK cells but not CD4^+^ or CD8^+^ T cells [59]. Cao et al. also showed that intratumoral injection of IL-18 significantly inhibited the growth of murine T241 fibrosarcomas, although the reported mechanism of action in this case was inhibition of tumor angiogenesis [31].

We have demonstrated in an immunocompetent orthotopic model of prostate cancer that intratumoral IL-18 has the ability to inhibit tumor growth. The anti-tumor effect is mediated by induction of IFN-γ expression by IL-18, resulting in stimulation of both the innate and adaptive immune. These results confirm that IL-18 holds promise as an immunotherapeutic agent for the treatment of prostate cancer.

## Supporting Information

Figure S1 **Characterisation of cell lines.** A) Activated splenic T cells were incubated with various concentrations of RM1-IL18 conditioned media and IFN-γ production was detected by ELISA. Increasing concentrations of CM induced IFN-γ production in a dose-dependent manner. B) RM1-IL18 tumors stained for IL-18 by immunohistochemistry (left panel); negative isotype control (right panel). C) As few as 400 RM1(BM)/IL-18lo-luc cells can be detected *in vitro* by bioluminescent imaging after addition of luciferin.
(TIF)

Figure S2 **Immunohistochemical staining for markers of apoptosis and cell proliferation in orthtopic RM1-IL18 tumours.** Representative images of tumours stained for caspase-3 (A & B) for apoptotic cells and Ki-67 (C & D) for proliferating cells is shown. E) The percentage of tumor sections (± standard error of mean) stained with the pro-apoptotic molecule caspase-3 (upper panel) and the proliferation-dependent protein Ki-67 (lower panel) was estimated after immunohistochemical staining of sections from multiple tumors. All treatments were compared to the Ctrl group using one-way ANOVA followed by Tukey's multiple comparison post test. Statistically significant p values are indicated for comparisons between treatment groups the corresponding Ctrl, * p value 0.01 to 0.05, ** p value 0.001 to 0.01, *** p<0.001.
(TIF)

Figure S3 **IL-18 increases tumor-infiltration of macrophages and neutrophils but not NK cells in C57BL/6 RAG1^-/-^ mice.** Histochemical and immunohistochemical staining of intraprostatic RM1-IL18 tumors from the RAG1^-/-^ mice described in the legend to Fig. 4 A, without (A, C & E) or with (B, D & F) the IL-18-neutralizing antibody. Immunohistochemical staining was performed with anti-F4/80 for macrophages (A & B) and anti-asialo-GM1 for NK cells (E & F). Histochemical staining (C & D) showing neutrophil infiltration indicated by green arrowheads is shown.
(TIF)

## References

[1] Parkin DM, Bray F, Ferlay J, Pisani P (2005). Global cancer statistics, 2002.. CA Cancer J Clin.

[2] Pienta KJ, Smith DC (2005). Advances in prostate cancer chemotherapy: a new era begins.. CA Cancer J Clin.

[3] Harris DT, Matyas GR, Gomella LG, Talor E, Winship MD (1999). Immunologic approaches to the treatment of prostate cancer.. Semin Oncol.

[4] Kowalczyk DW, Wysocki PJ, Mackiewicz A (2003). Cancer immunotherapy using cells modified with cytokine genes.. Acta Biochim Pol.

[5] Dunn GP, Old LJ, Schreiber RD (2004). The immunobiology of cancer immunosurveillance and immunoediting.. Immunity.

[6] Matsui K, Yoshimoto T, Tsutsui H, Hyodo Y, Hayashi N (1997). Propionibacterium acnes treatment diminishes CD4+ NK1.1+ T cells but induces type I T cells in the liver by induction of IL-12 and IL-18 production from Kupffer cells.. J Immunol.

[7] Okamura H, Tsutsi H, Komatsu T, Yutsudo M, Hakura A (1995). Cloning of a new cytokine that induces IFN-gamma production by T cells.. Nature.

[8] Stoll S, Jonuleit H, Schmitt E, Muller G, Yamauchi H (1998). Production of functional IL-18 by different subtypes of murine and human dendritic cells (DC): DC-derived IL-18 enhances IL-12-dependent Th1 development.. Eur J Immunol.

[9] Stoll S, Muller G, Kurimoto M, Saloga J, Tanimoto T (1997). Production of IL-18 (IFN-gamma-inducing factor) messenger RNA and functional protein by murine keratinocytes.. J Immunol.

[10] Pizarro TT, Michie MH, Bentz M, Woraratanadharm J, Smith MF (1999). IL-18, a novel immunoregulatory cytokine, is up-regulated in Crohn's disease: expression and localization in intestinal mucosal cells.. J Immunol.

[11] Udagawa N, Horwood NJ, Elliott J, Mackay A, Owens J (1997). Interleukin-18 (interferon-gamma-inducing factor) is produced by osteoblasts and acts via granulocyte/macrophage colony-stimulating factor and not via interferon-gamma to inhibit osteoclast formation.. J Exp Med.

[12] Conti B, Park LC, Calingasan NY, Kim Y, Kim H (1999). Cultures of astrocytes and microglia express interleukin 18.. Brain Res Mol Brain Res.

[13] Gracie JA, Robertson SE, McInnes IB (2003). Interleukin-18.. J Leukoc Biol.

[14] Gu Y, Kuida K, Tsutsui H, Ku G, Hsiao K (1997). Activation of interferon-gamma inducing factor mediated by interleukin-1beta converting enzyme.. Science.

[15] Ghayur T, Banerjee S, Hugunin M, Butler D, Herzog L (1997). Caspase-1 processes IFN-gamma-inducing factor and regulates LPS-induced IFN-gamma production.. Nature.

[16] Nakanishi K, Yoshimoto T, Tsutsui H, Okamura H (2001). Interleukin-18 is a unique cytokine that stimulates both Th1 and Th2 responses depending on its cytokine milieu.. Cytokine Growth Factor Rev.

[17] Kito T, Kuroda E, Yokota A, Yamashita U (2003). Cytotoxicity in glioma cells due to interleukin-12 and interleukin-18-stimulated macrophages mediated by interferon-gamma-regulated nitric oxide.. J Neurosurg.

[18] Leung BP, Culshaw S, Gracie JA, Hunter D, Canetti CA (2001). A role for IL-18 in neutrophil activation.. J Immunol.

[19] Schroder K, Hertzog PJ, Ravasi T, Hume DA (2004). Interferon-gamma: an overview of signals, mechanisms and functions.. J Leukoc Biol.

[20] Nagai H, Hara I, Horikawa T, Oka M, Kamidono S (2002). Gene transfer of secreted-type modified interleukin-18 gene to B16F10 melanoma cells suppresses in vivo tumor growth through inhibition of tumor vessel formation.. J Invest Dermatol.

[21] Tanaka F, Hashimoto W, Robbins PD, Lotze MT, Tahara H (2002). Therapeutic and specific antitumor immunity induced by co-administration of immature dendritic cells and adenoviral vector expressing biologically active IL-18.. Gene Ther.

[22] Yoshimura K, Hazama S, Iizuka N, Yoshino S, Yamamoto K (2001). Successful immunogene therapy using colon cancer cells (colon 26) transfected with plasmid vector containing mature interleukin-18 cDNA and the Igkappa leader sequence.. Cancer Gene Ther.

[23] Coughlin CM, Salhany KE, Wysocka M, Aruga E, Kurzawa H (1998). Interleukin-12 and interleukin-18 synergistically induce murine tumor regression which involves inhibition of angiogenesis.. J Clin Invest.

[24] Kishida T, Asada H, Satoh E, Tanaka S, Shinya M (2001). In vivo electroporation-mediated transfer of interleukin-12 and interleukin-18 genes induces significant antitumor effects against melanoma in mice.. Gene Ther.

[25] Hikosaka S, Hara I, Miyake H, Hara S, Kamidono S (2004). Antitumor effect of simultaneous transfer of interleukin-12 and interleukin-18 genes and its mechanism in a mouse bladder cancer model.. Int J Urol.

[26] Wang J, Kobayashi Y, Sato A, Kobayashi E, Murakami T (2004). Synergistic anti-tumor effect by combinatorial gene-gun therapy using IL-23 and IL-18 cDNA.. J Dermatol Sci.

[27] Micallef MJ, Yoshida K, Kawai S, Hanaya T, Kohno K (1997). In vivo antitumor effects of murine interferon-gamma-inducing factor/interleukin-18 in mice bearing syngeneic Meth A sarcoma malignant ascites.. Cancer Immunol Immunother.

[28] Okamoto T, Yamada N, Tsujimura T, Sugihara A, Nishizawa Y (2004). Inhibition by interleukin-18 of the growth of Dunn osteosarcoma cells.. J Interferon Cytokine Res.

[29] Subleski JJ, Hall VL, Back TC, Ortaldo JR, Wiltrout RH (2006). Enhanced antitumor response by divergent modulation of natural killer and natural killer T cells in the liver.. Cancer Res.

[30] Osaki T, Peron JM, Cai Q, Okamura H, Robbins PD (1998). IFN-gamma-inducing factor/IL-18 administration mediates IFN-gamma- and IL-12-independent antitumor effects.. J Immunol.

[31] Cao R, Farnebo J, Kurimoto M, Cao Y (1999). Interleukin-18 acts as an angiogenesis and tumor suppressor.. Faseb J.

[32] Redlinger RE, Mailliard RB, Lotze MT, Barksdale EM (2003). Synergistic interleukin-18 and low-dose interleukin-2 promote regression of established murine neuroblastoma in vivo.. J Pediatr Surg.

[33] Xia D, Zheng S, Zhang W, He L, Wang Q (2003). Effective induction of therapeutic antitumor immunity by dendritic cells coexpressing interleukin-18 and tumor antigen.. J Mol Med.

[34] Ju DW, Tao Q, Lou G, Bai M, He L (2001). Interleukin 18 transfection enhances antitumor immunity induced by dendritic cell-tumor cell conjugates.. Cancer Res.

[35] Tatsumi T, Gambotto A, Robbins PD, Storkus WJ (2002). Interleukin 18 gene transfer expands the repertoire of antitumor Th1-type immunity elicited by dendritic cell-based vaccines in association with therapeutic efficacy.. Cancer Res.

[36] Ju DW, Yang Y, Tao Q, Song WG, He L (2000). Interleukin-18 gene transfer increases antitumor effects of suicide gene therapy through efficient induction of antitumor immunity.. Gene Ther.

[37] Thompson TC, Southgate J, Kitchener G, Land H (1989). Multistage carcinogenesis induced by ras and myc oncogenes in a reconstituted organ.. Cell.

[38] Lochner M, Wagner H, Classen M, Forster I (2002). Generation of neutralizing mouse anti-mouse IL-18 antibodies for inhibition of inflammatory responses in vivo.. J Immunol Methods.

[39] Power CA, Pwint H, Chan J, Cho J, Yu Y (2009). A novel model of bone-metastatic prostate cancer in immunocompetent mice.. Prostate.

[40] Martiniello-Wilks R, Tsatralis T, Russell P, Brookes DE, Zandvliet D (2002). Transcription-targeted gene therapy for androgen-independent prostate cancer.. Cancer Gene Ther.

[41] Giedraitis V, He B, Huang WX, Hillert J (2001). Cloning and mutation analysis of the human IL-18 promoter: a possible role of polymorphisms in expression regulation.. J Neuroimmunol.

[42] Liu W, Tang Q, Jiang H, Ding X, Liu Y (2009). Promoter polymorphism of interleukin-18 in angiographically proven coronary artery disease.. Angiology.

[43] Nong LG, Luo B, Zhang L, Nong HB (2009). Interleukin-18 gene promoter polymorphism and the risk of nasopharyngeal carcinoma in a Chinese population.. DNA Cell Biol.

[44] Wei YS, Lan Y, Liu YG, Tang H, Tang RG (2007). Interleukin-18 gene promoter polymorphisms and the risk of esophageal squamous cell carcinoma.. Acta Oncol.

[45] Lebel-Binay S, Thiounn N, De Pinieux G, Vieillefond A, Debre B (2003). IL-18 is produced by prostate cancer cells and secreted in response to interferons.. Int J Cancer.

[46] Robertson MJ, Mier JW, Logan T, Atkins M, Koon H (2006). Clinical and biological effects of recombinant human interleukin-18 administered by intravenous infusion to patients with advanced cancer.. Clin Cancer Res.

[47] Robertson MJ, Kirkwood JM, Logan TF, Koch KM, Kathman S (2008). A dose-escalation study of recombinant human interleukin-18 using two different schedules of administration in patients with cancer.. Clin Cancer Res.

[48] Tarhini AA, Millward M, Mainwaring P, Kefford R, Logan T (2009). A phase 2, randomized study of SB-485232, rhIL-18, in patients with previously untreated metastatic melanoma.. Cancer.

[49] van Herpen CM, Looman M, Zonneveld M, Scharenborg N, de Wilde PC (2004). Intratumoral administration of recombinant human interleukin 12 in head and neck squamous cell carcinoma patients elicits a T-helper 1 profile in the locoregional lymph nodes.. Clin Cancer Res.

[50] Khorana AA, Rosenblatt JD, Sahasrabudhe DM, Evans T, Ladrigan M (2003). A phase I trial of immunotherapy with intratumoral adenovirus-interferon-gamma (TG1041) in patients with malignant melanoma.. Cancer Gene Ther.

[51] Mastrangelo MJ, Maguire HC, Eisenlohr LC, Laughlin CE, Monken CE (1999). Intratumoral recombinant GM-CSF-encoding virus as gene therapy in patients with cutaneous melanoma.. Cancer Gene Ther.

[52] Sangro B, Mazzolini G, Ruiz J, Herraiz M, Quiroga J (2004). Phase I trial of intratumoral injection of an adenovirus encoding interleukin-12 for advanced digestive tumors.. J Clin Oncol.

[53] Bursuker I, North RJ (1984). Generation and decay of the immune response to a progressive fibrosarcoma. II. Failure to demonstrate postexcision immunity after the onset of T cell-mediated suppression of immunity.. J Exp Med.

[54] North RJ, Bursuker I (1984). Generation and decay of the immune response to a progressive fibrosarcoma. I. Ly-1+2- suppressor T cells down-regulate the generation of Ly-1-2+ effector T cells.. J Exp Med.

[55] Komai-Koma M, Gracie JA, Wei XQ, Xu D, Thomson N (2003). Chemoattraction of human T cells by IL-18.. J Immunol.

[56] Gutzmer R, Langer K, Mommert S, Wittmann M, Kapp A (2003). Human dendritic cells express the IL-18R and are chemoattracted to IL-18.. J Immunol.

[57] Morel JC, Park CC, Kumar P, Koch AE (2001). Interleukin-18 induces rheumatoid arthritis synovial fibroblast CXC chemokine production through NFkappaB activation.. Lab Invest.

[58] Di Carlo E, Forni G, Lollini P, Colombo MP, Modesti A (2001). The intriguing role of polymorphonuclear neutrophils in antitumor reactions.. Blood.

[59] Kikuchi T, Akasaki Y, Joki T, Abe T, Kurimoto M (2000). Antitumor activity of interleukin-18 on mouse glioma cells.. J Immunother.

