# Supplementary figures and images for: IL-18 Inhibits Growth of Murine Orthotopic Prostate Carcinomas via Both Adaptive and Innate Immune Mechanisms
Source: PLoS One. 2011 Sep 15;6(9):e24241. doi: 10.1371/journal.pone.0024241 (PMC3174151; doi:10.1371/journal.pone.0024241)

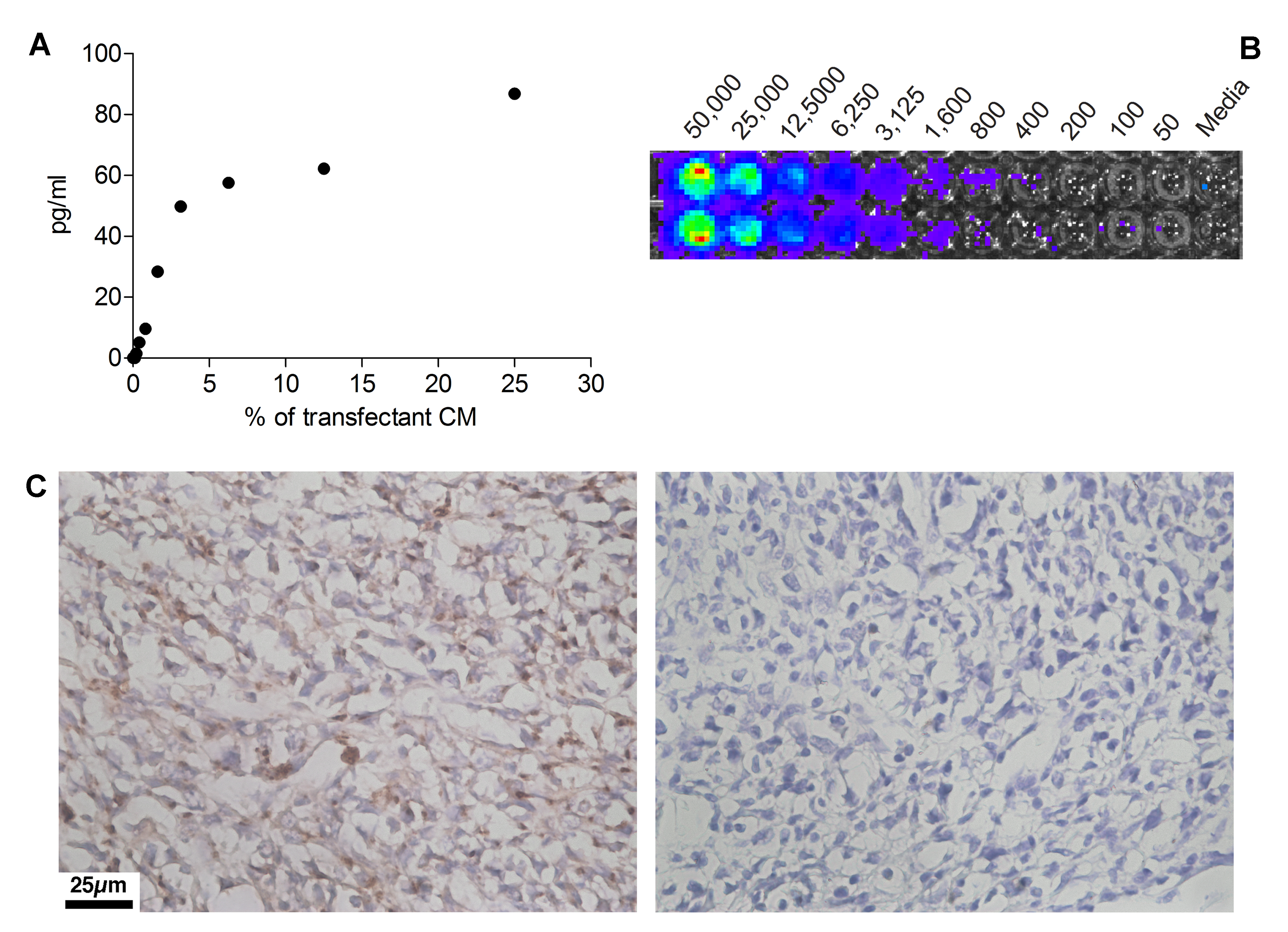

Supplement: Figure S1 — Characterisation of cell lines. A) Activated splenic T cells were incubated with various concentrations of RM1-IL18 conditioned media and IFN-γ production was detected by ELISA. Increasing concentrations of CM induced IFN-γ production in a dose-dependent manner. B) RM1-IL18 tumors stained for IL-18 by immunohistochemistry (left panel); negative isotype control (right panel). C) As few as 400 RM1(BM)/IL-18lo-luc cells can be detected in vitro by bioluminescent imaging after addition of luciferin. (TIF) [file pone.0024241.s001.tif]

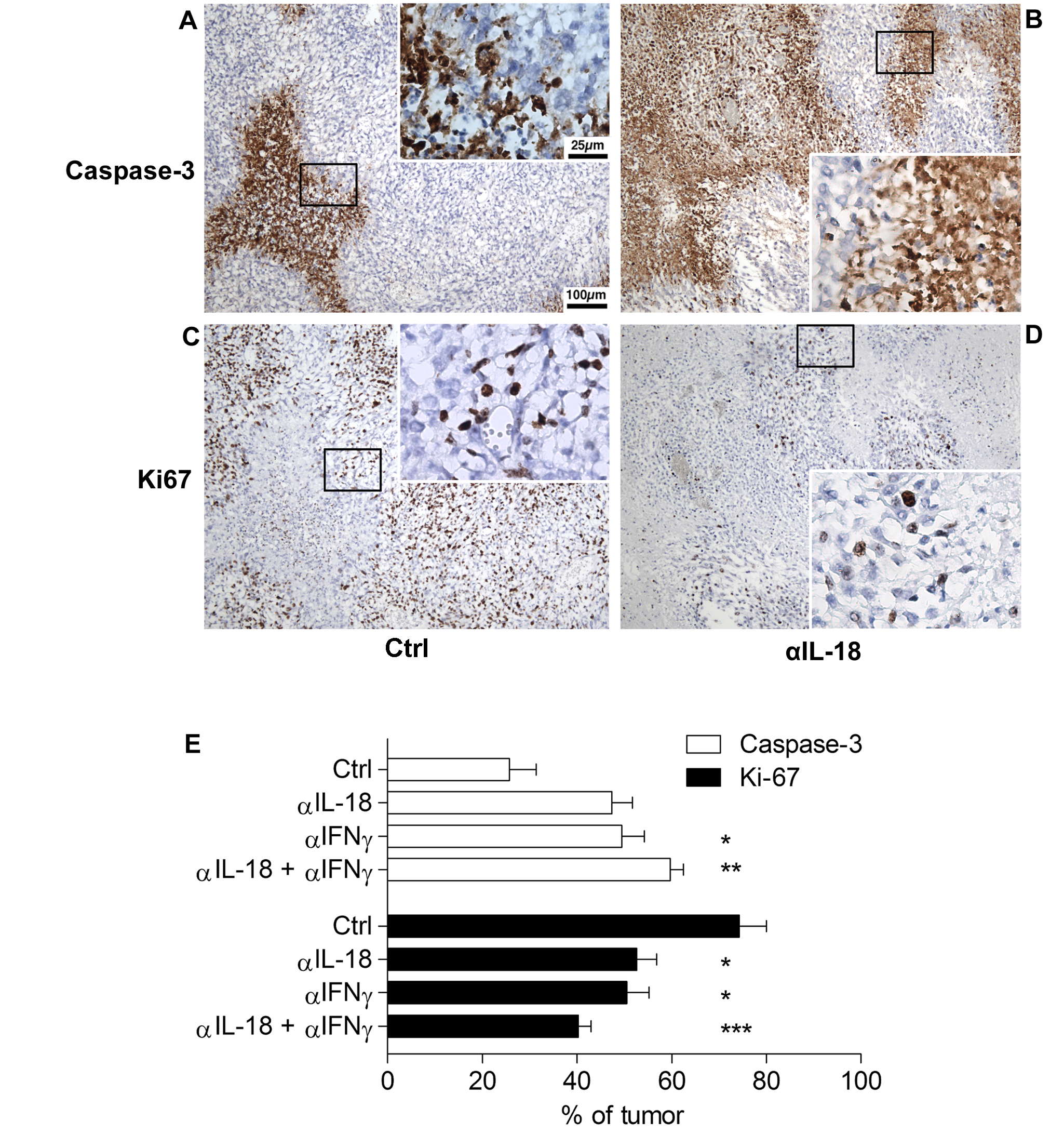

Supplement: Figure S2 — Immunohistochemical staining for markers of apoptosis and cell proliferation in orthtopic RM1-IL18 tumours. Representative images of tumours stained for caspase-3 (A & B) for apoptotic cells and Ki-67 (C & D) for proliferating cells is shown. E) The percentage of tumor sections (± standard error of mean) stained with the pro-apoptotic molecule caspase-3 (upper panel) and the proliferation-dependent protein Ki-67 (lower panel) was estimated after immunohistochemical staining of sections from multiple tumors. All treatments were compared to the Ctrl group using one-way ANOVA followed by Tukey's multiple comparison post test. Statistically significant p values are indicated for comparisons between treatment groups the corresponding Ctrl, * p value 0.01 to 0.05, ** p value 0.001 to 0.01, *** p<0.001. (TIF) [file pone.0024241.s002.tif]

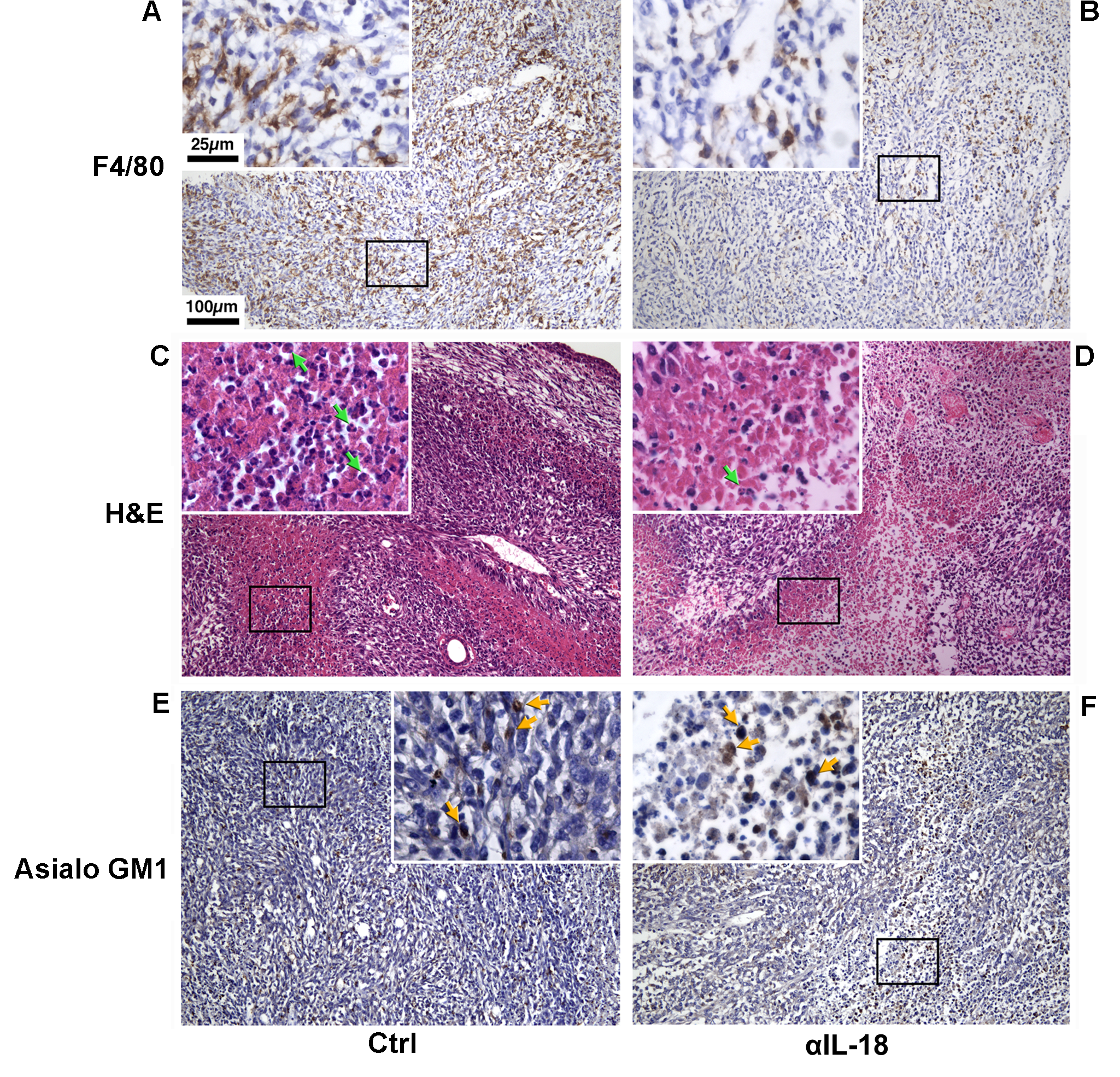

Supplement: Figure S3 — IL-18 increases tumor-infiltration of macrophages and neutrophils but not NK cells in C57BL/6 RAG1-/- mice. Histochemical and immunohistochemical staining of intraprostatic RM1-IL18 tumors from the RAG1-/- mice described in the legend to Fig. 4 A, without (A, C & E) or with (B, D & F) the IL-18-neutralizing antibody. Immunohistochemical staining was performed with anti-F4/80 for macrophages (A & B) and anti-asialo-GM1 for NK cells (E & F). Histochemical staining (C & D) showing neutrophil infiltration indicated by green arrowheads is shown. (TIF) [file pone.0024241.s003.tif]
